# The Establishment of Pseudorandom Ecological Microexpression Recognition Test (PREMERT) and Its Relevant Resting-State Brain Activity

**DOI:** 10.3389/fnhum.2020.00281

**Published:** 2020-07-30

**Authors:** Jianxin Zhang, Ming Yin, Deming Shu, Dianzhi Liu

**Affiliations:** ^1^School of Humanities, Jiangnan University, Wuxi, China; ^2^Jiangsu Police Institute, Nanjing, China; ^3^School of Education, Soochow University, Suzhou, China

**Keywords:** PREMERT, microexpression M, microexpression SD, eyes-open and eyes-closed resting states, ALFF difference

## Abstract

The EMERT (ecological microexpression recognition test) by Zhang et al. (2017) used between-subjects Latin square block design for backgrounds; therefore, participants could not get comparable scores. The current study used within-subject pseudorandom design for backgrounds to improve EMERT to PREMERT (pseudorandom EMERT) and used eyes-closed and eyes-open resting-state functional magnetic resonance imaging to detect relevant brain activity of PREMERT for the first time. The results showed (1) two new recapitulative indexes of PREMERT were adopted, such as microexpression M and microexpression SD. Using pseudorandom design, the participants could effectively identify almost all the microexpressions, and each microexpression type had significant background effect. The PREMERT had good split-half reliability, parallel-forms reliability, criterion validity, and ecological validity. Therefore, it could stably and efficiently detect the participants’ microexpression recognition abilities. Because of its pseudorandom design, all participants did the same test; their scores could be compared with each other. (2) amplitude of low-frequency fluctuations (ALFF; 0.01–0.1 Hz) in both eyes-closed and eyes-open resting states and ALFF difference could predict microexpression M, and the ALFF difference was less predictive. The relevant resting-state brain areas of microexpression M were some frontal lobes, insula, cingulate cortex, hippocampus, amygdala, fusiform gyrus, parietal lobe, caudate nucleus, precuneus, thalamus, putamen, temporal lobe, and cerebellum. (3) ALFFs in both eyes-closed and eyes-open resting states and ALFF difference could predict microexpression SD, and the ALFF difference was more predictive. The relevant resting-state brain areas of microexpression SD were some frontal lobes, central anterior gyrus, supplementary motor area, insula, hippocampus, amygdala, cuneus, occipital lobe, fusiform gyrus, parietal lobe, caudate nucleus, pallidum, putamen, thalamus, temporal lobe, and cerebellum. (4) There were many similar relevant resting-state brain areas, such as brain areas of expression recognition, microexpressions consciousness and attention, and the change from expression backgrounds to microexpression, and some different relevant resting-state brain areas, such as precuneus, insula, and pallidum, between microexpression M and SD. The ALFF difference was more sensitive to PREMERT fluctuations.

## Introduction

### The Ecological Microexpression Recognition Test

Microexpressions are very transitory expressions lasting about 1/25 to 1/2 s, which can reveal people’s true emotions they try to hide or suppress (Ekman and Friesen, 1975; Porter et al., 2012). Matsumoto et al. (2000) developed the Japanese and Caucasian Brief Affect Recognition Test (JACBART, classical microexpression recognition) to measure microexpression recognition. The participants would first see a neutral expression for 2,000 ms, and then a microexpression was presented for a little time, followed by the neutral expression for 2,000 ms again. Participants needed to check out the microexpression type. The neutral expression backgrounds could eliminate the visual aftereffects of the microexpression. But it did not examine the influence of backgrounds with emotional expressions. Therefore, Zhang et al. (2014) for the first time explored the background effects on microexpressions and found that when backgrounds were negative (sadness), all microexpressions (anger, disgust, fear, surprise, and happiness) recognition accuracies were significantly lower than those under positive (happiness) or neutral backgrounds; when the backgrounds and the microexpressions were consistent in property (negative or positive), microexpression recognition accuracies were significantly lower than those when they were inconsistent in property. The research has broken through the JACBART paradigm. Although it was very instructive, it needed to be further developed. (1) It did not explore either all backgrounds or all microexpressions. (2) It did not either reveal that the microexpressions in different backgrounds were ecological microexpressions or set up ecological microexpression recognition test to test reliability and validity.

Yin et al. (2016) proposed that all basic expression kinds for both backgrounds and microexpressions needed to be detected to set up the ecological microexpression recognition test. Therefore, Zhang et al. (2017) examined the recognition characteristics of six basic expression kinds of microexpressions (sadness, fear, anger, disgust, surprise, happiness) under seven basic expression kinds of backgrounds (the six basic expressions and neutral) to establish an ecological microexpression recognition test (EMERT) and found that the test had good retest reliability, criterion validity, and ecological validity: (1) the ecological microexpression recognition was generally significantly related to the JACBART microexpression recognition and common expression recognition. (2) The background’s main effect of sadness, fear, anger, and disgust microexpressions were significant; the background’s main effect of surprise and happiness microexpressions were not significant, but there was a wide difference between them with the common expressions. (3) The ecological microexpression recognition had stable fluctuation. Zhu et al. (2017) used simplified edition of EMERT to find microexpression recognition difference between depressive patients and normal people. Yin et al. (2019) extended EMERT to WEMERT (weak ecological microexpression recognition test), which was another type of EMERT.

However, the current study found that in all the EMERTs between-subjects Latin square block design was used for backgrounds, although within-subject pseudorandom design was used for microexpressions. There were seven blocks, in each of which only one background type was set, but all six kinds of microexpressions were repeated 10 times pseudorandomly. To balance exercise effect and fatigue effect, the blocks order was different with Latin square design among participants. For example, one participant did all microexpression recognition under sadness background in the first block, but another participant did it in the seventh block. Of course, the two participants’ recognition scores of microexpressions under sadness background could be added up to balance the exercise effect and fatigue effect, but we could not compare the scores between the two participants because the exercise effect and fatigue effect between them were different. The two participants did actually different EMERTs.

Therefore, we will use the within-subject pseudorandom design for backgrounds in the current study rather than Latin square block design for backgrounds in EMERT. For example, in one trial in the current study, the expression backgrounds were sadness; in the next trial, the backgrounds would stay sadness or change to other expressions. In each block, there were almost all kinds of microexpressions under all kinds of expression backgrounds, and their order was pseudorandom, so their exercise effects and fatigue effects were almost equal. Most importantly, each participant did the same test and could get comparable test scores. Then, by the within-subject pseudorandom design, EMERT will be improved to PREMERT (pseudorandom EMERT).

### Brain Activation of Ecological Microexpression Recognition

There were few published researches detecting brain activation of ecological microexpression recognition. Shen (2012) in Xiaolan Fu’s team used functional near-infrared spectroscopy to find that the brain area responsible for classical microexpression recognition was in the left frontal lobe, whereas the brain area responsible for expression recognition was in right frontal lobe. Zhang (2014) in Xiaolan Fu’s team used functional magnetic resonance imaging (fMRI) to find that, for anger and neutral microexpressions, activation of inferior parietal lobule was induced more in negative expression backgrounds than in neutral expression backgrounds, whereas activation of right precuneus was induced more in positive expression backgrounds than in neutral expression backgrounds. For happiness microexpressions, activation of parahippocampal gyrus was induced more in positive backgrounds. These studies revealed the brain mechanisms of classical microexpressions and three-ecological-microexpression recognition, but more brain mechanisms of more ecological microexpression recognition need further research.

As there were 36 ecological microexpressions in EMERT (Zhang et al., 2017), it is obviously neither feasible nor economical to adopt task-state fMRI. Resting-state fMRI is a viable and economical option. Resting-state fMRI investigates spontaneous activity or functional connections within the brain at rest. If a certain cognitive task is associated with certain brain areas that are active in a resting state, then these brain areas are associated with the cognitive task. If brain areas whose activity in resting state related to two cognitive tasks differ, then the brain mechanisms underlying execution of these two cognitive tasks are different. The rationales are as follows: in the resting state, participants do not perform any cognitive task, so the spontaneous activity of a brain area is its baseline activity and its functional strength index. If it is related to a cognitive task, it indicates the brain area is related to the cognitive task, which is like that if the density and the neurons number of a brain area are related to a cognitive task, the brain area is related to the cognitive task. The rationales are generally accepted and used by resting-state researchers (Heuvel and Pol, 2010; Li et al., 2016; Liu et al., 2017; Jiang et al., 2018), and the related resting-state brain areas of a cognitive task are usually proved to be its task-state brain areas using task-state fMRI, which is, when the participants perform the cognitive task, its related resting-state brain areas are usually activated (This result also holds true for the current study, see section “Results” and “Discussion”). Therefore, brain spontaneous activity in the resting state is a stable index to measure the individual cognitive characteristics (Liu et al., 2017). One of the classic indexes is the amplitude of low-frequency fluctuations (ALFF, 0.01–0.1 Hz) value, including most of the psychological cognitive process. The higher and lower amplitudes are background noise such as physiological activity. There were eyes-closed and eyes-open resting states. Nakano et al. (2012) and Nakano (2015) found that, in eyes-closed, subjects focused on internal feeling and self-consciousness, whereas in eyes-open, subjects turned to external stimulus processing, and the transition from eyes-closed to eyes-open was from internal feeling and self-consciousness to external stimulus processing. However, resting-state fMRI was not used in any microexpression research. Therefore, resting-state fMRI needs to be used in microexpression research.

### Improvements Made in the Current Study

The current study used pseudorandom design for backgrounds to improve EMERT to PREMERT (pseudorandom EMERT). Therefore, all participants did the same PREMERT, and each of them could get comparable test scores. The current study for the first time used eyes-closed and eyes-open resting-state fMRI to detect relevant resting-state brain activity of PREMERT. We assume that (1) PREMERT has good reliability and validity to measure participants’ comparable ecological microexpression recognition ability. (2) Related brain areas of microexpression M in resting state can reveal different neural mechanism of different microexpression recognition. (3) Related brain areas of microexpression SD in resting state can reveal neural mechanism of the expression background effect on microexpression recognition. The microexpression SD was used as the background effect on microexpression recognition (Zhang et al., 2017), see section “Behavioral Data.”

## Materials and Methods

### Participants

Fifty-three college students participated in the study. Males and females were 24 and 29. The age mean (M) ± SD = 21.60 ± 2.39. They were all right-handed with normal or corrected-to-normal eyesight and without color blindness. They all volunteered and could quit at any time. Each participant completed an informed consent form before the experiments. They got corresponding rewards after completing the experiments. The experiments were in accordance with the ethical guidelines of the Declaration of Helsinki and were approved by the Scientific Review Committee of Faculty of Psychology, Southwest University, China.

### Experimental Apparatus and Materials

Seven kinds of basic expression opened mouth pictures of eight models (four male and four female participants, including white, black, and yellow people) from the NimStim face expression database (Tottenham et al., 2009) were used as the backgrounds, namely, neutral, sadness, fear, anger, disgust, surprise, and happiness. Except neutral expressions, the other six kinds of expressions were used as microexpressions. The pixels of all images were modified to be 338 × 434 with gray background (GRB: 127, 127, 127) (Zhang et al., 2017). The Lenovo ThinkPad T410i notebook computer and 14.1-inch LCD display screen, which had 1,280 × 800 of resolution and 60 Hz of refresh rate, were used to do the experiments. E-prime 2.0 was used to compile the experimental procedure.

### Experimental Design and Procedures

The experiment was 7 (expression backgrounds: neutral vs. sadness vs. fear vs. anger vs. disgust vs. surprise vs. happiness) × 6 (microexpressions: sadness vs. fear vs. anger vs. disgust vs. surprise vs. happiness) within subject design. As there were seven types of expression backgrounds, in order to balance the sequential effect, the within-subject pseudorandom design for backgrounds was used in the current study rather than Latin square block design for backgrounds in EMERT (Zhang et al., 2017), and the within-subject pseudorandom design for microexpressions was also used in the current study as in EMERT. For example, in one trial in the current study, the backgrounds were sadness; in the next trial, the backgrounds would stay sadness or change to other expressions. In each block, there were almost all 42 kinds of microexpressions (six kinds of microexpressions under seven-expression background), and each of the 42 kinds of microexpressions repeated eight times pseudorandomly in all six blocks throughout the test, so their exercise effects and fatigue effects were almost equal by putting all six blocks together. Most importantly, each participants did the same test and could get comparable test scores. Therefore, the current study improved EMERT (Zhang et al., 2017) to PREMERT (pseudorandom EMERT).

Participants were 70 cm away from the screen. On the computer keyboard, six keys of SDF-JKL corresponded with “sadness,” “fear,” “anger,” “disgust,” “surprise,” and “happiness.” Before the experiment, the participants were asked to put the ring finger, middle finger, and index finger of their left hands on the SDF keys, respectively, whereas the index finger, middle finger, and ring finger of their right hands on JKL keys. And then they did key pressing practice. First, one of the six kinds of expressions words (except neutral) was presented 1,000 ms, and then six labels “sadness, fear, anger, disgust, surprise, happiness” with “S, D, F, J, K, L” under the corresponding labels appeared on the screen, and the participants needed to recognize the expression word and press the right key as accurately as possible. There were 30 trials, and six kinds of expression words were pseudorandomly presented for five times. Because the key pressing practice was very simple, all participants did it well with greater than 90% accuracy.

**FIGURE 1:**
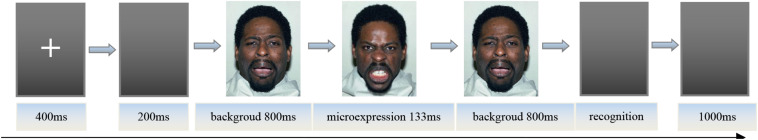
The picture of experiment procedure.

Note: These images are licensed by the copyright owner, Tottenham et al.

After the key pressing practice was completed, the instructor informed the participants of the procedure. First, the center of the screen would show the “+” for 400 ms; second, the empty screen lasted 200 ms, and then the front background expression image was presented for 800 ms, after which the microexpression image would appear for 133 ms, followed by 800 ms of back background expression image (Matsumoto et al., 2000; Zhang et al., 2017). The front and back backgrounds and microexpressions were of the same model’s face, and the front and back backgrounds were the same. Participants needed to try to identify the briefly presented microexpression between front and back backgrounds. Later, six labels “sadness, fear, anger, disgust, surprise, happiness” with “S, D, F, J, K, L” under the corresponding labels appeared on the screen. The participants were asked to press a key according to the microexpression they saw as accurately as possible instead of as soon as possible (no time limit). After the participants pressed the key, an empty screen would show for 1,000 ms. Then the fixation point “+” was presented for 400 ms, and the next trial started. The experiment procedure is shown in Figure 1.

The participants practiced the experimental procedure after understanding the instructions. There were a total of 14 trials, of which seven kinds of backgrounds appeared two times, and six kinds of microexpressions each appeared two to three times. The participants were asked to determine the type of microexpressions. After the experimental procedure practice was completed, they started a formal trial. In order to allow the participants to get enough rest, the experiment was divided into six blocks. Rest between each two blocks was 1 min. The experiment had 7 (backgrounds) × 6 (microexpressions) × 8 (models) = 336 trials.

A month before the PREMERT, the participants needed to do two EMERT measurements. The two EMERT measurements were similar with the PREMERT, except that they used Latin square block design for grounds and that they were performed on a PC (Lenovo LX-GJ556D) with a 17-inch color display (resolution 1,024 × 768, refresh rate 60 Hz). Because Zhang et al. (2017) proved EMERT had good reliability and validity, we used the correlation between PREMERT and EMERT as the parallel-forms reliability and criterion validity of PREMERT.

### Resting-State Data Collection and Analysis

The fMRI data were collected using a Siemens 3.0-T MRI scanner and an eight-channel phased front head coil. Eyes-closed and eyes-open resting-state imaging used gradient echo single-excitation echo-planar imaging. Scan parameters were as follows: repetition time (TR) = 2,000 ms, echo time (TE) = 30 ms, flip angle (FA) = 90°, field of view (FOV) = 220 × 220 mm^2^, matrix size = 64 × 64 mm^2^, depth = 3 mm, planar resolution = 3.13 × 3.13 mm^2^, interval scanning, 33 layers, layer spacing = 0.6 mm, total 240 layers. Structural imaging used a three-dimensional T1-weighted imaging (MP-RAGE) sequence with sagittal scans. Scan parameters were the following: TR = 2,600 ms, TE = 3.02 ms, FA = 8°, no interval, FOV = 256 × 256 mm^2^, matrix size = 256 × 256 mm^2^, total 176 layers. All the participants first received the structural scan, and then half received the eyes-closed and eyes-open resting-state scans, and half received the eyes-open and eyes-closed resting-state scans.

Pretreatment and analysis of resting-state data used DPARSF 3.0 Advanced Edition Calculate (Yan et al., 2016) in Original Space (Warp by DARTEL), following standard procedures: (1) conversion of raw DICOM-format data to NIFTI format. To allow for signal stabilization of the image, the first 10 TR images were removed, after which time layer correction (slice timing) and head movement correction (realignment, adopting Friston 24) were conducted. If head movement greater than 2 mm occurred during resting state, the data were deleted. (2) The new segment + DARTEL was used to split the structural T1 data without standardization and register the T1 split data directly to the resting-state functional images. Before registration of structural and functional data, the AC-PC line of each participant’s T1 image and the resting-state function was registered, and then automatic registration was applied. Therefore, the resting-state analysis took place in the original T1 space. (3) Head motion, linear drift, white matter, and cerebrospinal fluid via regression were adjusted for. (4) Amplitude of low-frequency fluctuations (filter range, 0.01–0.1 Hz) were calculated. (5) The resting-state function was registered to the standard MNI space (normalization), using a 3 × 3 × 3 mm^3^ voxel size, with 4 × 4 × 4 mm^3^ full width at half maximum smoothing.

REST1.8 (Song et al., 2011) was first used to extract the ALFFs during resting states in 116 anatomical automatic labeling (AAL) brain areas. Second, SPSS19.0 was used to implement correlation analyses between ALFFs in 116 AAL brain areas and the scores of PREMERT. The ALFF difference of eyes-open minus eyes-closed was used as an index of transition from internal feeling and self-consciousness to external stimulus processing. Its significance was detected by correlation analyses between it and the scores of PREMERT.

## Results

SPSS 19.0 was used for statistics. There were 53 valid participants in PREMERT, 46 valid participants in eyes-closed resting state, and 51 valid participants in eyes-open resting state. Seven participants’ head movements were greater than 2 mm in eyes-closed resting state, and two participants in eyes-open resting state.

### Behavioral Data

The accuracy and standard deviation of each microexpression/expression in PREMERT is shown in Table 1. A 7 (background expressions) × 6 (microexpressions) analysis of variance was conducted. Background expressions and microexpressions were independent variables within-subject. (1) Sphericity test of background expressions showed the variance was homogeneous, *p* > 0.05. The main effect of background expressions was significant, *F*(6,53) = 14.61, *p* < 0.001, η_p_^2^ = 0.219, which meant that background expressions affected microexpressions. (2) Sphericity test of microexpressions showed the variance was homogeneous, *p* > 0.05. The main effect of microexpressions was significant, *F*(5,53) = 84.22, *p* < 0.001, η_p_^2^ = 0.618, which meant that microexpression recognition was different. (3) Sphericity test of backgrounds × microexpressions showed the variance was not homogeneous, *p* < 0.05, and then we performed Greenhouse correction and found that background expressions and microexpressions had significant interaction effect, *F*(14.16,53) = 15.26, *p* < 0.001, η_p_^2^ = 0.227, which meant that background expressions and microexpressions influenced each other. Those results indicated that the ecological validity of PREMERT was good that it could detect the differences among different microexpressions and among different expression backgrounds (Zhang et al., 2017; Yin et al., 2019).

**TABLE 1:** The scores of PREMERT.

Microexpressions	Mean ± SD (*n* = 53)	*t*	Cohen *d*
Sadness	0.66 ± 0.27	13.1***	1.83
Fear	0.51 ± 0.27	9.14***	1.27
Anger	0.65 ±c0.21	16.26***	2.30
Disgust	0.63 ± 0.29	11.46***	1.60
Surprise	0.74 ± 0.27	15.48***	2.12
Happiness	0.87 ± 0.26	19.49***	2.71
Sadness under fear	0.34 ± 0.24	5.20***	0.72
Sadness under anger	0.25 ± 0.25	2.40***	0.33
Sadness under disgust	0.28 ± 0.25	3.18***	0.45
Sadness under neutral	0.29 ± 0.24	3.86***	0.51
Sadness under surprise	0.41 ± 0.25	7.15***	0.97
Sadness under happiness	0.33 ± 0.27	4.54***	0.6
Fear under sadness	0.26 ± 0.19	3.75***	0.49
Fear under anger	0.29 ± 0.22	3.86***	0.56
Fear under disgust	0.31 ± 0.19	5.38***	0.75
Fear under neutral	0.31 ± 0.23	4.49***	0.62
Fear under surprise	0.17 ± 0.19	0.3	–
Fear under happiness	0.35 ± 0.25	5.33***	0.73
Anger under sadness	0.59 ± 0.32	9.60***	1.32
Anger under fear	0.60 ± 0.30	10.54***	1.44
Anger under disgust	0.43 ± 0.22	8.68***	1.2
Anger under neutral	0.63 ± 0.27	12.64***	1.72
Anger under surprise	0.61 ± 0.33	9.66***	1.34
Anger under happiness	0.49 ± 0.29	7.96***	1.11
Disgust under sadness	0.50 ± 0.24	10.10***	1.39
Disgust under fear	0.50 ± 0.24	9.95***	1.39
Disgust under anger	0.37 ± 0.26	5.56***	0.78
Disgust under neutral	0.60 ± 0.27	11.86***	1.6
Disgust under surprise	0.44 ± 0.25	8.04***	1.09
Disgust under happiness	0.52 ± 0.24	10.71***	1.47
Surprise under sadness	0.67 ± 0.23	15.54***	2.19
Surprise under fear	0.66 ± 0.23	15.39***	2.14
Surprise under anger	0.64 ± 0.27	12.76***	1.75
Surprise under disgust	0.56 ± 0.26	11.16***	1.51
Surprise under neutral	0.79 ± 0.25	17.9***	2.49
Surprise under happiness	0.73 ± 0.25	16.18***	2.25
Happiness under sadness	0.81 ± 0.25	18.83***	2.57
Happiness under fear	0.76 ± 0.28	15.41***	2.12
Happiness under anger	0.78 ± 0.3	14.66***	2.04
Happiness under disgust	0.74 ± 0.31	13.43***	1.85
Happiness under neutral	0.83 ± 0.26	18.78***	2.55
Happiness under surprise	0.76 ± 0.3	14.24***	1.98

***p < 0.001.

Because the participants have six keys to choose for each trial, the random level is 1/6. A single-sample *t* test was made for each microexpression recognition accuracy with random level 1/6, and it was found that almost all the microexpression recognition accuracies were significantly greater than random (*p*’s < 0.001), except that fear under surprise was not (*p* > 0.05). Because each microexpression/expression was compared only once to random level 1/6, there was no multiple comparisons, and no multiple comparison correction was required.

It is obvious that the PREMERT indexes were too many to be recapitulative enough for both participants and researchers. Then the mean of accuracy rates of a microexpression type under six backgrounds (except the same expression grounds as the microexpression, because in that case it was a normal expression rather than a microexpression) was used as the index of this microexpression type recognition, and it was abbreviated as microexpression M. The standard deviation of accuracy rates of this microexpression type under six backgrounds (except the same expression grounds as the microexpression) was used as the background effect index of this microexpression type recognition, which was called the fluctuations of the microexpression type recognition (Zhang et al., 2017; Yin et al., 2019), and it was abbreviated as microexpression SD. Therefore, we obtained two new recapitulative indexes of PREMERT. A single-sample *t* test was made for each microexpression M with random level 1/6, and it was found that all were significantly greater than random (*p*’s < 0.001). A single-sample *t* test was made for each microexpression SD with random level 0, and it was found that all were significantly greater than random (*p*’s < 0.001).

PREMERT was divided into odd PREMERT and even PREMERT, and their microexpression M’s and microexpression SDs were calculated, respectively. Then the Pearson correlation between odd PREMERT and even PREMERT was calculated as the split-half reliability. It was found that each microexpression M in odd PREMERT was significantly positively related to the corresponding one in even PREMERT, and the *r*’s were high; except anger SD, each microexpression SD in odd PREMERT was significantly positively related to the corresponding one in even PREMERT, and the *r*’s were either medium or high. Those results indicated that the split-half reliability of PREMERT was good. Because microexpression under neutral was microexpression in JACBART, and microexpression under the same expression background was expression, we used Pearson correlation between PREMERT and JACBART or expression as criterion validity (Zhang et al., 2017; Yin et al., 2019). It was found that each microexpression M in PREMERT was significantly positively related to the corresponding one in JACBART, and the *r*’s were high: sadness M, anger M, disgust M, and happiness M in PREMERT were significantly positively related to their corresponding expressions, and the *r*’s were medium. Those results indicated that the criterion validity of PREMERT was good. The new indexes of PREMERT, the indexes of odd PREMERT and even PREMERT, the indexes of JACBART, and expressions and their statistical results are shown in Table 2.

**TABLE 2:** The new scores of PREMERT and its split-half reliability and criterion validity.

Micro expression	PREMERT Mean ± SD (*n* = 53)	*t*	Cohen’s *d*	Odd PREMERT Mean ± SD (*n* = 53)	Even PREMERT Mean ± SD (*n* = 53)	*r*_O–E_	JACBART Mean ± SD (*n* = 53)	*r*_PR–J_	Expression Mean ± SD (*n* = 53)	*r*_PR–E_
SadnessM	0.32 ± 0.21	5.42***	0.73	0.30 ± 0.20	0.33 ± 0.22	0.85**	0.29 ± 0.24	0.75**	0.66 ± 0.27	0.31*
Fear M	0.28 ± 0.15	5.55***	0.76	0.26 ± 0.15	0.30 ± 0.18	0.72**	0.31 ± 0.23	0.76**	0.51 ± 0.27	–
Anger M	0.56 ± 0.26	11.12***	1.51	0.57 ± 0.25	0.55 ± 0.28	0.91**	0.63 ± 0.27	0.82**	0.65 ± 0.21	0.59**
Disgust M	0.49 ± 0.21	11.42***	1.54	0.46 ± 0.20	0.52 ± 0.24	0.78**	0.60 ± 0.27	0.87**	0.63 ± 0.29	0.57**
Surprise M	0.68 ± 0.2	18.59***	2.57	0.68 ± 0.21	0.67 ± 0.20	0.91**	0.79 ± 0.25	0.80**	0.74 ± 0.27	–
Happiness M	0.78 ± 0.26	17.23***	2.36	0.8 ± 0.24	0.76 ± 0.29	0.95**	0.83 ± 0.26	0.82**	0.87 ± 0.26	0.47**
Sadness SD	0.15 ± 0.06	17.65***	2.50	0.20 ± 0.08	0.21 ± 0.10	0.57**	–	–	–	–
Fear SD	0.16 ± 0.06	18.70***	2.67	0.20 ± 0.07	0.21 ± 0.08	0.38**	–	–	–	–
Anger SD	0.16 ± 0.06	19.89***	2.67	0.24 ± 0.08	0.19 ± 0.07	–	–	–	–	–
Disgust SD	0.17 ± 0.05	22.71***	3.40	0.21 ± 0.08	0.22 ± 0.08	0.33*	–	–	–	–
Surprise SD	0.16 ± 0.08	15.60***	2.00	0.22 ± 0.09	0.21 ± 0.09	0.34*	–	–	–	–
Happiness SD	0.10 ± 0.08	8.60***	1.25	0.14 ± 0.12	0.13 ± 0.11	0.70**	–	–	–	–

r_O–E_ was the r between odd PREMERT and even PREMERT. r_PR–J_ was the r between PREMERT and JACBART. r_PR–E_ was the r between PREMERT and expression. *p < 0.05, **p < 0.01, ***p < 0.001.

As such, we also obtained new recapitulative indexes of the two EMERT. Pearson correlation was made between PREMERT and EMERT, and it was found that each microexpression M in PREMERT was significantly positively related to the corresponding one in two EMERTs, and the *r*’s were either high or medium; anger SD, disgust SD, and happiness SD in PREMERT were significantly positively related to the corresponding microexpression SDs in two EMERT, and the *r*’s were either medium or low. Those results indicated that the parallel-forms reliability and criterion validity of PREMERT were good. The new indexes of PREMERT and EMERT and their statistical results are shown in Table 3.

**TABLE 3:** The parallel-forms reliability and criterion validity of PREMERT.

Microexpression	PREMERT Mean ± SD (n = 53)	First PREMERT Mean ± SD (n = 53)	*r*_*first*_	Second PREMERT Mean ± SD (n = 53)	*r*_*second*_
Sadness M	0.32 ± 0.21	0.36 ± 0.20	0.61***	0.45 ± 0.24	0.75***
Fear M	0.28 ± 0.15	0.30 ± 0.13	0.59***	0.31 ± 0.18	0.63***
Anger M	0.56 ± 0.26	0.69 ± 0.22	0.62***	0.71 ± 0.21	0.69***
Disgust M	0.49 ± 0.21	0.50 ± 0.19	0.70***	0.60 ± 0.20	0.78***
Surprise M	0.68 ± 0.2	0.73 ± 0.21	0.72***	0.71 ± 0.23	0.68***
Happiness M	0.78 ± 0.26	0.89 ± 0.23	0.62***	0.91 ± 0.17	0.71***
Sadness SD	0.15 ± 0.06	0.17 ± 0.07	–	0.16 ± 0.06	–
Fear SD	0.16 ± 0.06	0.18 ± 0.07	–	0.18 ± 0.07	–
Anger SD	0.16 ± 0.06	0.17 ± 0.07	0.35**	0.15 ± 0.09	0.36**
Disgust SD	0.17 ± 0.05	0.15 ± 0.06	0.29*	0.14 ± 0.06	0.35**
Surprise SD	0.16 ± 0.08	0.15 ± 0.08	–	0.15 ± 0.07	–
Happiness SD	0.10 ± 0.08	0.08 ± 0.09	0.54***	0.07 ± 0.09	0.47***

r_frist_ was the r between PREMERT and first EMERT. r’_s__econd_ was the r between PREMERT and second EMERT. *p < 0.05, **p < 0.01, ***p < 0.001.

To sum up, PREMERT established in the current study had good split-half reliability, parallel-forms reliability, criterion validity, and ecological validity. Therefore, it could stably and efficiently detect the participants’ microexpression recognition abilities. Because of its pseudorandom design, all participants did the same test; their scores could be compared with each other.

### Brain Imaging Data

**FIGURE 2:**
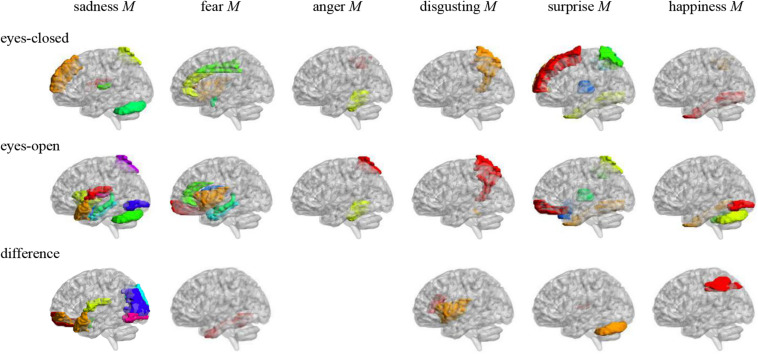
Anatomical automatic labeling brain areas whose ALFFs in eyes-closed and eyes-open resting-state and ALFF difference were related to the microexpression M. The brain areas were visualized with the BrainNet Viewer (http://www.nitrc.org/projects/bnv/) (Xia et al., 2013), the same below.

Pearson correlation analysis was made between ALFFs of resting-state and microexpression M (Table 4 and Figure 2). (1) In the eyes-closed resting state, ALFFs in frontal lobe, insula, cingulate cortex, amygdala, fusiform gyrus, parietal lobe, precuneus, thalamus, temporal lobe, and cerebellum were significantly correlated with some microexpression M. (2) In the eyes-open resting state, ALFFs in frontal lobe, insula, cingulate cortex, hippocampus, amygdala, fusiform gyrus, parietal lobe, caudate nucleus, precuneus, thalamus, temporal lobe, and cerebellum were significantly correlated with some microexpression M. (3) In the ALFF difference of eyes-open minus eyes-closed resting states, ALFF difference in frontal lobe, insula, parietal lobe, putamen, temporal lobe, and cerebellum were significantly correlated with some microexpression M.

**TABLE 4:** The *r*’s between ALFFs of resting-state and microexpression M.

Resting-state	AAL brain area	ALFF (Mean ± SD)	Sadness M	Fear M	Anger M	Disgust M	Surprise M	Happiness M
Eyes-closed	Frontal_Sup_L	0.86 ± 0.05					0.32*	
Eyes-closed	Rolandic_Oper_R	0.86 ± 0.03	0.35*	0.39**				
Eyes-closed	Frontal_Sup_Medial_L	0.96 ± 0.07	0.29*					
Eyes-closed	Frontal_Sup_Medial_R	0.93 ± 0.07					0.33*	
Eyes-closed	Insula_R	0.97 ± 0.05		0.40**				
Eyes-closed	Cingulum_Ant_L	0.99 ± 0.06		0.35*				
Eyes-closed	Cingulum_Mid_L	0.94 ± 0.03		0.30*				
Eyes-closed	Amygdala_L	1.09 ± 0.11		0.31*				
Eyes-closed	Fusiform_R	0.87 ± 0.02					−0.31*	−0.33*
Eyes-closed	Parietal_Sup_L	0.97 ± 0.07					0.34*	
Eyes-closed	Parietal_Sup_R	0.93 ± 0.06	0.33*				0.45**	
Eyes-closed	Parietal_Inf_R	1.06 ± 0.06			0.32*	0.30*	0.39**	0.32*
Eyes-closed	Precuneus_L	1.08 ± 0.06				0.30*		
Eyes-closed	Thalamus_L	1.01 ± 0.08					−0.39**	
Eyes-closed	Thalamus_R	1.01 ± 0.08					−0.31*	
Eyes-closed	Heschl_L	1.01 ± 0.07	0.30*					
Eyes-closed	Heschl_R	1.08 ± 0.1		0.30*				
Eyes-closed	Cerebelum_Crus1_L	0.97 ± 0.1	−0.31*					
Eyes-closed	Cerebelum_3_L	1.63 ± 0.27			0.33*			
Eyes-closed	Cerebelum_4_5_L	1.19 ± 0.09			0.37*			
Eyes-closed	Vermis_3	1.59 ± 0.21			0.30*			
Eyes-open	Frontal_Sup_Orb_L	0.86 ± 0.07					−0.30*	
Eyes-open	Frontal_Sup_Orb_R	0.83 ± 0.07		−0.29*				
Eyes-open	Rolandic_Oper_L	0.84 ± 0.03	0.32*					
Eyes-open	Rolandic_Oper_R	0.86 ± 0.03	0.46**					
Eyes-open	Insula_L	0.92 ± 0.03	0.40**	0.36**				
Eyes-open	Insula_R	0.97 ± 0.04	0.45**	0.52**				
Eyes-open	Cingulum_Ant_L	0.99 ± 0.06		0.33*				
Eyes-open	Hippocampus_L	0.93 ± 0.04	0.35*	0.31*				
Eyes-open	ParaHippocampal_L	1.12 ± 0.08	0.39**	0.31*				
Eyes-open	Amygdala_L	1.11 ± 0.11	0.29*					
Eyes-open	Occipital_Inf_L	0.94 ± 0.06	−0.31*					−0.28*
Eyes-open	Fusiform_R	0.87 ± 0.03					−0.28*	−0.35*
Eyes-open	Parietal_Sup_R	0.93 ± 0.06	0.35*		0.29*		0.42**	
Eyes-open	Parietal_Inf_R	1.05 ± 0.06					0.35*	
Eyes-open	Precuneus_L	1.07 ± 0.06				0.29*		
Eyes-open	Caudate_L	0.95 ± 0.08		0.33*				
Eyes-open	Thalamus_L	0.99 ± 0.08					−0.39**	
Eyes-open	Thalamus_R	0.99 ± 0.07					−0.33*	
Eyes-open	Heschl_L	1 ± 0.07	0.36**					
Eyes-open	Heschl_R	1.05 ± 0.08	0.34*	0.31*				
Eyes-open	Temporal_Pole_Sup_L	1.09 ± 0.11	0.36**					
Eyes-open	Temporal_Pole_Sup_R	1.01 ± 0.08	0.28*				0.38**	
Eyes-open	Cerebelum_Crus1_L	0.95 ± 0.1	−0.30*					−0.34*
Eyes-open	Cerebelum_3_L	1.65 ± 0.26			0.35*			
Eyes-open	Cerebelum_4_5_L	1.18 ± 0.08			0.30*			
Eyes-open	Cerebelum_6_R	0.92 ± 0.05						−0.41**
Eyes-open	Vermis_1_2	1.88 ± 0.37				0.28*		
Eyes-open	Vermis_3	1.58 ± 0.21			0.29*			
Difference	Frontal_Inf_Tri_R	0.01 ± 0.03				0.31*		
Difference	Insula_L	0.01 ± 0.03				0.35*		
Difference	Insula_R	0.01 ± 0.02				0.32*		
Difference	Parietal_Inf_L	−0.01 ± 0.03						−0.30*
Difference	SupraMarginal_R	−0.01 ± 0.03				0.35*		
Difference	Heschl_R	−0.03 ± 0.06					0.30*	
Difference	Temporal_Pole_Sup_L	0.01 ± 0.05	0.33*					
Difference	Temporal_Pole_Sup_R	0 ± 0.05	0.30*					
Difference	Temporal_Inf_R	0.01 ± 0.02		0.34*				
Difference	Cerebelum_Crus1_L	−0.02 ± 0.05					−0.33*	
Difference	Cerebelum_Crus2_R	−0.01 ± 0.09	−0.33*					

*p < 0.05, **p < 0.01.

**FIGURE 3:**
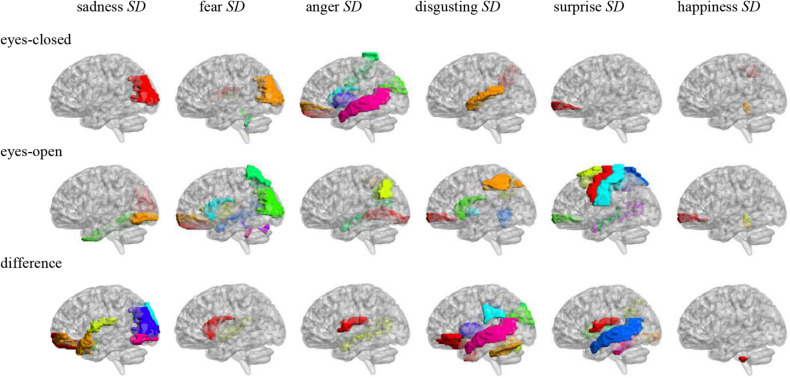
Anatomical automatic labeling brain areas whose ALFFs in eyes-closed and eyes-open resting-state and ALFF difference were related to the microexpression SD.

Pearson correlation analysis was made between ALFFs of resting-state and microexpression SD (Table 5 and Figure 3). (1) In the eyes-closed resting state, ALFFs in frontal lobe, hippocampus, occipital lobe, parietal lobe, caudate nucleus, pallidum, temporal lobe, and cerebellum were significantly correlated with some microexpression SD. (2) In the eyes-open resting state, ALFFs in central anterior gyrus, frontal lobe, supplementary motor area, insula, hippocampus, occipital lobe, fusiform gyrus, parietal lobe, caudate nucleus, pallidus, temporal lobe, and cerebellum were significantly correlated with some microexpression SD. (3) In the ALFF difference of eyes-open minus eyes-closed resting states, ALFF differences in frontal lobe, hippocampus, amygdala, wedge, occipital lobe, parietal lobe, caudate nucleus, putamen, thalamus, temporal lobe, and cerebellum were significantly correlated with some microexpression SD.

**TABLE 5:** The *r*’s between ALFFs of resting-state and microexpression SD.

Resting-state	AAL brain area	ALFF (mean ± sd)	Sadness SD	Fear SD	Anger SD	Disgust SD	Surprise SD	Happiness SD
Eyes-closed	Frontal_Sup_Orb_R	0.8 ± 0.06				0.30*		
Eyes-closed	Rolandic_Oper_R	0.86 ± 0.03		0.32*				
Eyes-closed	Frontal_Mid_Orb_L	0.98 ± 0.11				0.33*	0.30*	
Eyes-closed	Hippocampus_R	0.89 ± 0.04				0.34*		
Eyes-closed	Cuneus_L	1.13 ± 0.16				−0.33*		
Eyes-closed	Occipital_Mid_L	0.94 ± 0.06	0.32*	−0.32*				
Eyes-closed	Postcentral_R	0.88 ± 0.07				0.29*		
Eyes-closed	Parietal_Inf_R	1.06 ± 0.06						−0.37*
Eyes-closed	Angular_R	1.05 ± 0.07			−0.34*			
Eyes-closed	Caudate_L	0.95 ± 0.09				0.37*		
Eyes-closed	Caudate_R	0.87 ± 0.04				0.42**		
Eyes-closed	Putamen_L	0.79 ± 0.04				0.31*		
Eyes-closed	Pallidum_L	0.83 ± 0.04				0.37*		
Eyes-closed	Heschl_R	1.08 ± 0.1		0.36*				
Eyes-closed	Temporal_Sup_L	1.06 ± 0.05			0.31*			
Eyes-closed	Temporal_Mid_L	0.9 ± 0.03				0.29*		
Eyes-closed	Vermis_1_2	1.86 ± 0.34		0.35*				
Eyes-closed	Vermis_3	1.59 ± 0.21						−0.30*
Eyes-closed	Vermis_10	2.22 ± 0.56		0.31*				
Eyes-open	Precentral_L	0.82 ± 0.04					−0.40**	
Eyes-open	Precentral_R	0.85 ± 0.05					−0.29*	
Eyes-open	Frontal_Sup_Orb_R	0.83 ± 0.07		−0.29*				
Eyes-open	Supp_Motor_Area_L	1 ± 0.08					−0.28*	
Eyes-open	Frontal_Mid_Orb_R	0.93 ± 0.07		−0.31*		0.30*	0.30*	0.33*
Eyes-open	Insula_R	0.97 ± 0.04		0.29*				
Eyes-open	Hippocampus_R	0.9 ± 0.03					−0.28*	
Eyes-open	Lingual_R	1.01 ± 0.05			0.36**			
Eyes-open	Occipital_Mid_L	0.96 ± 0.05		−0.33*				
Eyes-open	Occipital_Mid_R	0.93 ± 0.04	0.29*					
Eyes-open	Occipital_Inf_L	0.94 ± 0.06	−0.30*					
Eyes-open	Occipital_Inf_R	0.94 ± 0.06	−0.35*					
Eyes-open	Fusiform_R	0.87 ± 0.03	−0.33*					
Eyes-open	Postcentral_L	0.8 ± 0.04					−0.32*	
Eyes-open	Parietal_Sup_L	0.97 ± 0.07		−0.29*				
Eyes-open	Parietal_Sup_R	0.93 ± 0.06					−0.30*	
Eyes-open	Parietal_Inf_L	0.98 ± 0.04				−0.28*		
Eyes-open	Parietal_Inf_R	1.05 ± 0.06			−0.29*		−0.30*	
Eyes-open	Angular_L	1.04 ± 0.06			−0.32*			
Eyes-open	Angular_R	1.06 ± 0.07			−0.31*	−0.29*		
Eyes-open	Caudate_L	0.95 ± 0.08		0.28*		0.30*		
Eyes-open	Caudate_R	0.88 ± 0.05				0.31*		
Eyes-open	Pallidum_L	0.84 ± 0.04				0.29*		
Eyes-open	Pallidum_R	0.83 ± 0.03					−0.29*	
Eyes-open	Temporal_Mid_R	0.98 ± 0.03		−0.29*	−0.31*			
Eyes-open	Vermis_1_2	1.88 ± 0.37		0.31*				−0.29*
Eyes-open	Vermis_3	1.58 ± 0.21						−0.31*
Eyes-open	Vermis_4_5	1.18 ± 0.13				0.28*		
Eyes-open	Vermis_7	0.82 ± 0.06		−0.28*				
Eyes-open	Vermis_10	2.19 ± 0.57		0.29*				
Difference	Frontal_Sup_Orb_L	0.03 ± 0.07	0.29*					
Difference	Frontal_Mid_Orb_L	0.04 ± 0.08	0.35*					
Difference	Frontal_Inf_Orb_L	0.02 ± 0.03				−0.30*		
Difference	Rolandic_Oper_L	0 ± 0.02	0.37*		−0.31*		−0.43**	
Difference	Rolandic_Oper_R	0 ± 0.02			−0.43**			
Difference	Olfactory_L	0.01 ± 0.04				−0.30*		
Difference	Hippocampus_R	0.01 ± 0.02				−0.37*		
Difference	Amygdala_R	0.01 ± 0.04	0.32*					
Difference	Cuneus_L	-0.06 ± 0.11				0.36*		
Difference	Cuneus_R	-0.06 ± 0.1	−0.30*			0.29*		
Difference	Occipital_Sup_L	-0.01 ± 0.06	−0.33*					
Difference	Occipital_Sup_R	0 ± 0.05	−0.38*					
Difference	Occipital_Mid_L	0.02 ± 0.04	−0.48**					
Difference	Occipital_Mid_R	0.02 ± 0.04	−0.37*					
Difference	Occipital_Inf_L	0.02 ± 0.05	−0.41**					
Difference	Occipital_Inf_R	0.02 ± 0.06	−0.37*				0.33*	
Difference	SupraMarginal_L	0 ± 0.03				−0.40**		
Difference	SupraMarginal_R	-0.01 ± 0.03				−0.32*		
Difference	Angular_R	0.01 ± 0.05					−0.30*	
Difference	Caudate_L	0.01 ± 0.04		0.35*				
Difference	Caudate_R	0.01 ± 0.02		0.34*				
Difference	Putamen_L	0.02 ± 0.02				−0.43**		
Difference	Putamen_R	0.01 ± 0.02				−0.34*	−0.29*	
Difference	Thalamus_L	−0.01 ± 0.03					0.30*	
Difference	Thalamus_R	−0.01 ± 0.04					0.33*	
Difference	Temporal_Sup_R	−0.03 ± 0.04		−0.33*				
Difference	Temporal_Pole_Sup_L	0.01 ± 0.05	0.31*					
Difference	Temporal_Mid_L	0 ± 0.02				−0.34*	−0.34*	
Difference	Temporal_Mid_R	−0.01 ± 0.03			−0.39**			
Difference	Temporal_Inf_R	0.01 ± 0.02				−0.40**		
Difference	Cerebelum_3_L	0.03 ± 0.15					0.32*	
Difference	Cerebelum_3_R	0.03 ± 0.18					0.38**	
Difference	Cerebelum_4_5_L	−0.01 ± 0.05					0.46**	
Difference	Cerebelum_4_5_R	−0.02 ± 0.05					0.33*	
Difference	Cerebelum_6_L	−0.02 ± 0.05				0.31*		
Difference	Cerebelum_10_L	0.04 ± 0.21						0.30*
Difference	Vermis_3	0 ± 0.13					0.49**	
Difference	Vermis_4_5	−0.03 ± 0.07					0.39**	
Difference	Vermis_7	0.01 ± 0.03				−0.39**		

*p < 0.05, **p < 0.01.

## Discussion

### The PREMERT Had Good Reliability and Validity

Pseudorandom design for backgrounds was used in the current study to improve EMERT to PREMERT (pseudorandom EMERT), and two new recapitulative indexes of PREMERT such as microexpression M and microexpression SD were used. Almost all the microexpression recognition accuracy rates and all the microexpression M’s were significantly greater than random, which meant that the participants could effectively identify almost all the microexpressions. Only fear under surprise was not significantly greater than random, which might be because the fear microexpression and the surprise backgrounds had similar face muscle status. All the microexpression SDs were significantly greater than random, which meant that each microexpression type had significant background effect (Zhang et al., 2017; Yin et al., 2019).

The variance analysis showed that the main effect of expression backgrounds and microexpressions and their interaction effect were significant, which indicated that the ecological validity of PREMERT was good (Zhang et al., 2017; Yin et al., 2019).

Each microexpression M in odd PREMERT was significantly positively related to the corresponding one in even PREMERT, and the *r*’s were high; except anger SD, each microexpression SD in odd PREMERT was significantly positively related to the corresponding one in even PREMERT, and the *r*’s were either medium or high, which indicated that the split-half reliability of PREMERT was good. Each microexpression M in PREMERT was significantly positively related to the corresponding one in JACBART, and the *r*’s were high; sadness M, anger M, disgust M, and happiness M in PREMERT were significantly positively related to the corresponding expressions, and the *r*’s were medium, which indicated that the criterion validity of PREMERT was good.

Each microexpression M in PREMERT was significantly positively related to the corresponding one in the two EMERT, and the *r*’s were either high or medium; anger SD, disgust SD, and happiness SD in PREMERT were significantly positively related to the corresponding microexpression SDs in the two EMERT, and the *r*’s were either medium or low. The results showed that using two EMERT as duplicates and calibrations, the PREMERT had good parallel-forms reliability and calibration validity. But the pseudorandom design in PREMERT changed the sadness SD, fear SD, and surprise SD. Of course, because the PREMERT was 1 month after the two EMERTs, it could not detect the participants’ initial microexpression recognition ability, but it could detect the participants’ microexpression recognition ability after twice natural exposure exercises by the two EMERTs (Matsumoto et al., 2000; Hurley, 2012; Hurley et al., 2014). Therefore, the PREMERT and the two EMERTs were only approximate parallel forms of each other, but we obtained the split-half reliability and the criterion validity of PREMERT above, so the approximate parallel-forms reliability could serve as an enrichment and supplement.

In summary, the PREMERT established in the current study had good split-half reliability, parallel-forms reliability, criterion validity, and ecological validity. Therefore, it could stably and efficiently detect the participants’ microexpression recognition abilities. Because of its pseudorandom design, all participants did the same test; their scores could be compared with each other.

### The Relevant Resting-State Brain Areas of Microexpression M in PREMERT

In the eyes-closed resting state, ALFFs in frontal lobe, insula, cingulate cortex, amygdala, fusiform gyrus, parietal lobe, precuneus, thalamus, temporal lobe, and cerebellum were significantly correlated with some microexpression M, of which the insula, cingulate cortex, amygdala, fusiform gyrus, precuneus, thalamus, and temporal lobe were common brain areas of expression recognition (Hu et al., 2009); the frontal lobe, parietal lobe, precuneus, insula, cingulate, thalamus, and temporal lobe might be responsible for microexpression consciousness and attention (Dehaene et al., 2011; Huang et al., 2017), and the precuneus and cerebellum might be responsible for the change from expression backgrounds to microexpression (Penhune and Steele, 2012; Zhang, 2014), which of course need further research to determine, the same as below.

In the eyes-open resting state, ALFFs in frontal lobe, insula, cingulate cortex, hippocampus, amygdala, fusiform gyrus, parietal lobe, caudate nucleus, precuneus, thalamus, temporal lobe, and cerebellum were significantly correlated with some microexpression M, of which the insula, cingulate cortex, hippocampus, amygdala, fusiform gyrus, thalamus, and temporal lobe were common brain areas of expression recognition; the frontal lobe, parietal lobe, precuneus, insula, cingulate, hippocampus, thalamus and temporal lobe might be responsible for microexpressions consciousness and attention, and the caudate nucleus, precuneus, and cerebellum might be responsible for the change from expression backgrounds to microexpression. It can be seen that microexpression M was significantly correlated with similar brain areas in both eyes-closed and eyes-open resting states.

Amplitude of low-frequency fluctuation differences of eyes-open minus eyes-closed resting states in frontal lobe, insula, parietal lobe, putamen, temporal lobe, and cerebellum were significantly correlated with some microexpression M, of which the insula and temporal lobe were common expression recognition brain areas, the frontal lobe, parietal lobe, insula, and temporal lobe might be responsible for microexpressions consciousness and attention, and the putamen and cerebellum might be responsible for the change from expression backgrounds to microexpression. It can be seen that there were relatively fewer relevant resting-state brain areas in the ALFF difference, which were basically included by relevant resting-state brain areas in eyes-closed and eyes-open resting states, and the absolute values of *r*’s were smaller.

It was found that ALFFs in both eyes-closed and eyes-open resting states and ALFF difference could predict microexpression M, and the ALFF difference was less predictive and could not predict anger M. According to the relevant resting-state brain areas and logic, there might be three cognitive processes in ecological microexpression recognition, such as the expression recognition, microexpressions consciousness and attention, and the change from expression background to microexpression. Whether and when each of them occurs and whether some other cognitive processes exist by developing new behavioral measurement methods to separate them and by task-state fMRI and Event-Related Potential (ERP) need to be explored in the future. Nakano et al. (2012) and Nakano (2015) found that transition from eyes-closed to eyes-open was from internal feeling to external stimulus processing. However, no study has taken the ALFF difference as a quantitative sensitivity index from internal feeling to external stimulus, and no study has investigated its significance. In the current study, we defined the ALFF difference as the quantitative sensitivity index from internal feeling to external stimulus, and it was found that the ALFF difference could predict PREMERT, indicating significance.

Shen (2012) found that the brain area responsible for classical microexpression recognition was in the left frontal lobe, whereas the brain area responsible for expression recognition was in the right frontal lobe. In the current study, it was found that, for PREMERT, both the left and right frontal lobes and more brain areas were involved. Zhang (2014) found that, for anger and neutral microexpressions, activation of the inferior parietal lobule was induced more in the negative expression backgrounds than in the neutral expression backgrounds, whereas activation of the right precuneus was induced more in the positive expression backgrounds than in the neutral expression backgrounds. For happiness microexpressions, activation of the parahippocampal gyrus was induced more in the positive backgrounds. The current study also found that these brain areas were involved in PREMERT, and more brain areas were involved. There might be two reasons for these difference: (1) the PREMERT in the current study was more comprehensive and ecological, and there was more background effect; and (2) the correlation analysis of resting state was adopted in the current study, but the comparative analysis of task-states has been used in previous studies either between microexpressions and expressions or among different microexpressions; therefore, many common brain areas either of microexpressions and expressions or of different microexpressions might be ignored by statistics. Zhang et al. (2017) established EMERT, and Yin et al. (2019) established WEMERT, but they did not investigate the relevant resting-state brain areas. In the current study, PREMERT was established, and the relevant resting-state brain areas were comprehensively investigated. Of course, further researches are needed to determine which function brain areas are responsible for.

### The Relevant Resting-State Brain Areas of Microexpression SD in PREMERT

In the eyes-closed resting state, ALFFs in frontal lobe, hippocampus, occipital lobe, parietal lobe, caudate nucleus, pallidum, temporal lobe, and cerebellum were significantly correlated with some microexpression SD. In the eyes-open resting state, ALFFs in central anterior gyrus, frontal lobe, supplementary motor area, insula, hippocampus, occipital lobe, fusiform gyrus, parietal lobe, caudate nucleus, pallidum, temporal lobe, and cerebellum were significantly correlated with some microexpression SD. It can be seen that microexpression SD was significantly associated with similar brain areas in both eyes-closed and eyes-open resting states. However, in the eyes-open resting state, some motor areas were added in the relevant resting-state brain areas.

Amplitude of low-frequency fluctuation differences of eyes-open minus eyes-closed resting states in frontal lobe, hippocampus, amygdala, cuneus, occipital lobe, parietal lobe, caudate nucleus, putamen, thalamus, temporal lobe, and cerebellum were significantly correlated with some microexpression SD. It can be seen that there were more relevant resting-state brain areas in the ALFF difference, which basically included the relevant resting-state brain areas in eyes-closed and eyes-open resting states, but some expression recognition areas were added. The absolute value of *r*’s were larger.

It was found that ALFFs in both eyes-closed and eyes-open resting states and ALFF difference could predict microexpression SD, and the ALFF difference was more predictive, indicating significance.

In EMERT and WEMERT, Zhang et al. (2017) and Yin et al. (2019) defined the microexpression SD as the fluctuation of the ecological microexpression to quantify the background effect, but did not investigate the relevant resting-state brain areas. The current study comprehensively investigated the relevant resting-state brain areas involved in the quantification of the background effect. Of course, further researches are needed to determine which function brain areas are responsible for.

### The Similarities and Differences of the Relevant Resting-State Brain Areas of Microexpression M and SD

Microexpression M and SD were significantly correlated with similar brain areas in eyes-closed resting state, eyes-open resting states, and ALFF difference, such as brain areas of expression recognition, brain areas of microexpressions consciousness and attention, and brain areas of the change from expression backgrounds to microexpression. There were more relevant resting-state brain areas of microexpression M and SD in the eyes-open resting state than in the eyes-closed resting state. The ALFF difference predicted microexpression M worse, but predicted microexpression SD better than ALFFs in eyes-closed and eyes-open resting states, indicating that the ALFF difference was more sensitive to PREMERT fluctuations, which was consistent with the ALFF difference definition of the sensitivity from internal feelings to external stimulus.

### The Limitations and Prospects of This Study

The current study measured only the six-basic-microexpression recognition, but the recognition mechanism of composite microexpressions with mixed emotions that are combinations of the six basic emotions may be fundamentally different from them, which should be more ecological and more realistic (Blank et al., 2020). In the future, we can use ephemeral composite expressions with mixed emotions under expression background to approximate composite microexpressions. The first important step is to capture enough composite expressions. Blank et al. (2020) provided an effective method to get a composite expression with mostly sad and slightly angry in realistic scenes, which can be expanded to get more composite expressions.

Of course, a more ideal method is to directly obtain composite microexpressions through experiments or real scenes, but that would be difficult.

## Conclusion

The current study used within-subject pseudorandom design for backgrounds to improve EMERT to PREMERT (pseudorandom EMERT) and used eyes-closed and eyes-open resting-state fMRI to detect relevant resting-state brain activity of PREMERT. The results showed the following:

(1) Two new recapitulative indexes of PREMERT were adopted, such as microexpression M and microexpression SD. Using pseudorandom design, the participants could effectively identify almost all the microexpressions, and each microexpression type had significant background effect. The PREMERT had good split-half reliability, parallel-forms reliability, criterion validity, and ecological validity. Therefore, it could stably and efficiently detect the participants’ microexpression recognition abilities. Because of its pseudorandom design, all participants did the same test; their scores could be compared with each other.
(2) Amplitudes of low-frequency fluctuation in both eyes-closed and eyes-open resting states and ALFF difference could predict microexpression M, and the ALFF difference was less predictive. The relevant resting-state brain areas of microexpression M were some frontal lobes, insula, cingulate cortex, hippocampus, amygdala, fusiform gyrus, parietal lobe, caudate nucleus, precuneus, thalamus, putamen, temporal lobe, and cerebellum.
(3) Amplitudes of low-frequency fluctuation in both eyes-closed and eyes-open resting states and ALFF difference could predict microexpression SD, and the ALFF difference was more predictive. The relevant resting-state brain areas of microexpression SD were some frontal lobes, central anterior gyrus, supplementary motor area, insula, hippocampus, amygdala, cuneus, occipital lobe, fusiform gyrus, parietal lobe, caudate nucleus, pallidum, putamen, thalamus, temporal lobe, and cerebellum.
(4) There were many similarities and some differences of the relevant resting-state brain areas between microexpression M and SD. The ALFF difference was more sensitive to PREMERT fluctuations.

## Data Availability Statement

The datasets generated for this study are available on request to the corresponding authors.

## Ethics Statement

The studies involving human participants were reviewed and approved by the Scientific Review Committee of Faculty of Psychology, Southwest University, China. The patients/participants provided their written informed consent to participate in this study. Written informed consent was obtained from the individual(s) for the publication of any potentially identifiable images or data included in this article.

## Author Contributions

JZ provided main research ideas and financial support, and was responsible for research design, data collection and analysis, and manuscript writing. MY provided part of research ideas and financial support, and was responsible for research design, data analysis, and manuscript writing. DS was responsible for experiment programming with E-Prime and supporting experiment implementation. DL was responsible for guiding the design, implementation, data analysis and manuscript writing of the whole research, and provided financial support. All authors contributed to the article and approved the submitted version.

## Conflict of Interest

The authors declare that the research was conducted in the absence of any commercial or financial relationships that could be construed as a potential conflict of interest.

## References

[B1] Blank C. Zaman S. Wesley A. Tsiamyrtzis P. Da Cunha Silva D. R. Gutierrez-Osuna R. (2020). “Emotional footprints of email interruptions,” in *Proceedings of the 2020 CHI Conference on Human Factors in Computing Systems. CHI ’20*, Honolulu, HI: Association for Computing Machinery, 155.

[B2] Dehaene S. Changeux J. P. Naccache L. (2011). “The global neuronal workspace model of conscious access: from neuronal architectures to clinical applications,” in *Characterizing Consciousness: From Cognition to the Clinic?*, eds Dehaene S. Christen Y. (Berlin: Springer Berlin Heidelberg), 55–84. 10.1007/978-3-642-18015-6_4

[B3] Ekman P. Friesen W. V. (1975). *Unmasking the Face:A Guide to Recognising Theemotions From Facial Cues.* Englewood Cliffs, NJ: Prentice Hall.

[B4] Heuvel M. P. V. D. Pol H. E. H. (2010). Exploring the brain network: a review on resting-state fmri functional connectivity. *Eur. Neuropsychopharmacol.* 20 519–534. 10.1016/j.euroneuro.2010.03.008 20471808

[B5] Hu X. Q. Fu G. Y. Shi Z. Y. (2009). Review and prospect of mirror neuron system. *Adv. n Psychol. Sci.* 17 118–125.

[B6] Huang J. P. Li Y. L. Zhang J. X. Wang X. P. Huang C. L. Chen A. T. (2017). fMRI investigation on gradual change of awareness states in implicit sequence learning. *Sci. Rep.* 7 1–8. 29196661 10.1038/s41598-017-16340-2 PMC5711927

[B7] Hurley C. M. (2012). Do you see what I see? Learning to detect micro expressions of emotion. *Motiv. Emot.* 36 371–381. 10.1007/s11031-011-9257-2

[B8] Hurley C. M. Anker A. E. Frank M. G. Matsumoto D. Hwang H. C. (2014). Background factors predicting accuracy and improvement in micro expression recognition. *Motiv. Emot.* 38 700–714. 10.1007/s11031-014-9410-9

[B9] Jiang Q. Hou L. L. Qiu J. Li C. R. Wang H. Z. (2018). The relationship between the caudate nucleus-orbitomedial prefrontal cortex connectivity and reactive aggression: a resting-state fMRI study. *Acta Psychol. Sin.* 50 655–666.

[B10] Li W. F. Tong D. D. Qiu J. Zhang Q. L. (2016). The neural basis of scientific innovation problems solving. *Acta Psychol. Sin.* 48 331–342.

[B11] Liu J. Liao X. Xia M. He Y. (2017). Chronnectome fingerprinting: identifying individuals and predicting higher cognitive functions using dynamic brain connectivity patterns. *Hum. Brain Mapp.* 39 902–915. 10.1002/hbm.23890 29143409 PMC6866558

[B12] Matsumoto D. Leroux J. Wilsoncohn C. Raroque J. Kooken K. Ekman P. (2000). A new test to measure emotion recognition ability: matsumoto and ekman’s japanese and caucasian brief affect recognition test (JACBART). *J. Nonverbal Behav.* 24 179–209.

[B13] Nakano T. (2015). Blink-related dynamic switching between internal and external orienting networks while viewing videos. *Neurosci. Res.* 96 54–58. 10.1016/j.neures.2015.02.010 25828154

[B14] Nakano T. Kato M. Morito Y. Itoi S. Kitazawa S. (2012). From the cover: blink-related momentary activation of the default mode network while viewing videos. *Proc. Natl. Acad. Sci. U.S.A.* 110 702–706. 10.1073/pnas.1214804110 23267078 PMC3545766

[B15] Penhune V. B. Steele C. J. (2012). Parallel contributions of cerebellar, striatal and M1 mechanisms to motor sequence learning. *Behav. Brain Res.* 226 579–591. 10.1016/j.bbr.2011.09.044 22004979

[B16] Porter S. ten Brinke L. Wallace B. (2012). Secrets and lies: Involuntary leakage in deceptive facial expressions as a function of emotional intensity. *J. Nonverbal Behav.* 36 23–37. 10.1007/s10919-011-0120-7

[B17] Shen X. B. (2012). *The Temporal Characteristics and Mechanisms of Microexpression Recognizing.* Doctoral dissertation, Graduate University of Chinese Academy of Sciences, Beijing.

[B18] Song X. W. Dong Z. Y. Long X. Y. Li S. F. Zuo X. N. Zhu C. Z. (2011). REST: A toolkit for resting-state functional magnetic resonance imaging data processing. *PLoS One* 6:25031. 10.1371/journal.pone.0025031 21949842 PMC3176805

[B19] Tottenham N. Tanaka J. W. Leon A. C. Mccarry T. Nurse M. Hare T. A. (2009). The nimstim set of facial expressions: judgments from untrained research participants. *Psychiatry Res.* 168 242–249. 10.1016/j.psychres.2008.05.006 19564050 PMC3474329

[B20] Xia M. Wang J. He Y. (2013). brainnet viewer: a network visualization tool for human brain connectomics. *PLoS One* 8:e68910. 10.1371/journal.pone.0068910 23861951 PMC3701683

[B21] Yan C. G. Wang X. D. Zuo X. N. Zang Y. F. (2016). DPABI: data processing & analysis for (Resting-State) brain imaging. *Neuroinformatics* 14 339–351. 10.1007/s12021-016-9299-4 27075850

[B22] Yin M. Tian L. C. Hua W. Zhang J. X. Liu D. Z. (2019). The establishment of weak ecological microexpressions recognition test (WEMERT): an extension on EMERT. *Front. Psychol.* 10:275. 10.3389/fpsyg.2019.00275 30890973 PMC6411658

[B23] Yin M. Zhang J. X. Shi A. Q. Liu D. Z. (2016). Characteristics, recognition, training of microexpressions and their influence factors. *Adv. Psychol. Sci.* 24 1723–1736.

[B24] Zhang J. X. Lu L. Yin M. Zhu C. L. Huang C. L. Liu D. Z. (2017). The establishment of ecological microexpression recognition test (emert):an improvement on jacbart microexpression recognition test. *Acta Psychol. Sin.* 49 886–896.

[B25] Zhang M. (2014). *The Effect of Emotional Context on Micro-expression Recognition and Its Mechanism.* Doctoral dissertation, Graduate University of Chinese Academy of Sciences, Beijing.

[B26] Zhang M. Fu Q. Chen Y. H. Fu X. (2014). Emotional context influences micro-expression recognition. *PLoS One* 9:e95018. 10.1371/journal.pone.0095018 24736491 PMC3988169

[B27] Zhu C. L. Chen X. Y. Zhang J. X. Liu Z. Y. Tang Z. Xu Y. T. (2017). Comparison of ecological micro-expression recognition in patients with depression and healthy individuals. *Front. Behav. Neurosci.* 11:119. 10.3389/fnbeh.2017.00199 29089879 PMC5651037

